# ROR2 knockdown suppresses breast cancer growth through PI3K/ATK signaling

**DOI:** 10.18632/aging.103400

**Published:** 2020-07-02

**Authors:** Muhong Guo, Ge Ma, Xiaolan Zhang, Weiwei Tang, Junfeng Shi, Qian Wang, Ye Cheng, Bin Zhang, Jin Xu

**Affiliations:** 1 Department of General Surgery, Nanjing First Hospital, Nanjing Medical University, Nanjing, China; 2 Department of Breast Surgery, The First Affiliated Hospital with Nanjing Medical University, Nanjing, China; 3 Department of Breast Surgery, The Affiliated Changzhou No.2 People's Hospital of Nanjing Medical University, Changzhou, China; 4 Department of Oncology, Nanjing First Hospital, Nanjing Medical University, Nanjing, China

**Keywords:** ROR2, breast cancer cell, proliferation, apoptosis, PI3K/AKT signaling

## Abstract

The receptor tyrosine kinase like orphan receptor 2 (ROR2) has been implicated in the pathogenesis of a variety of human cancers, including breast cancer. Here, we analyzed the clinical significance of ROR2 in breast cancer (BC) progression, and its function in the regulation of BC cell proliferation and growth. Analysis of *ROR2* mRNA levels in 45 BC tissues and adjacent non-tumor tissues revealed that *ROR2* expression was significantly increased in BC tissues, and that it correlated with tumor diameter. Kaplan-Meier disease-free survival (DFS) analysis demonstrated that BC patients with higher *ROR2* expression had lower DFS. Knockdown of ROR2 suppressed *in vitro* proliferation of BC cells and promoted apoptosis, while ROR2 overexpression induced BC cell proliferation and suppressed apoptosis. Importantly, ROR2 suppression also reduced the tumor growth in mouse BC xenografts, indicating that ROR2 promotes BC tumorigenesis *in vivo*. In addition, our data revealed that ROR2 promotes proliferation of BC cells by activating the PI3K/AKT signaling pathway. Together, our results indicate that ROR2 acts as an oncogenic gene in breast cancer, and suggest that the ROR2/PI3K/AKT regulatory network contributes to breast cancer progression.

## INTRODUCTION

Breast cancer (BC) is one of the most common cancers in women [1]. The receptor tyrosine kinases (RTKs) family plays an important role in physiological processes and development, but also in cancer progression. Receptor tyrosine kinase-like orphan receptor (ROR) forms a small subfamily within the RTK; it has a conserved domain structure consisting of two members, ROR1 and ROR2 [2]. ROR2 is thought to be a crucial regulator in human cancers, serving as a tumor-inducible protein and an oncogene [3]. ROR2 expression is often dysregulated in human cancers, and increased ROR2 levels promote tumor growth, migration, and invasion in multiple cancers including hepatocellular carcinoma [4], non-small cell lung cancer [5], cervical cancer [6], pancreatic cancer [7], melanoma [8, 9], colon cancer [10], head and neck squamous cell carcinoma [11], and breast cancer [12]. A previous Kaplan-Meier survival analysis has indicated that ROR2 is an independent prognostic factor for squamous carcinoma and gallbladder adenocarcinoma, and that low ROR2 levels inhibit squamous carcinoma and gallbladder adenocarcinoma growth [13]. The ROR2 expression is also increased in human non-small cell lung cancer (NSCLC) and correlates with an advanced TNM stage, indicating that ROR2 might be an independent prognostic factor in NSCLC [5]. In multiple myeloma, ROR2 has been shown to exert a pivotal role in cancer cell adhesion; genomic studies have indicated that the pathways mostly deregulated by ROR2 are phosphatidylinositol 3-kinase (PI3K)/AKT and mTOR [14]. However, very little is known about the expression and function of ROR2 in breast cancer.

In this study, we analyzed the clinical significance of ROR2 in BC progression, and its function in regulating BC cell proliferation and growth. Our data demonstrate that high ROR2 levels correlate with poor clinical outcomes in BC patients. In addition, our results show that ROR2 induces BC cell proliferation and tumor growth *in vitro* and *in vivo*, and that the mechanism involves activation of the PI3K/ATK signaling pathway. Together, our data suggest that ROR2 may represent a novel indicator of poor prognosis in BC patients, and might serve as a potential diagnostic biomarker and therapeutic target in breast cancer.

## RESULTS

### High ROR2 levels correlate with poor clinical outcomes in BC patients

**Figure 1:**
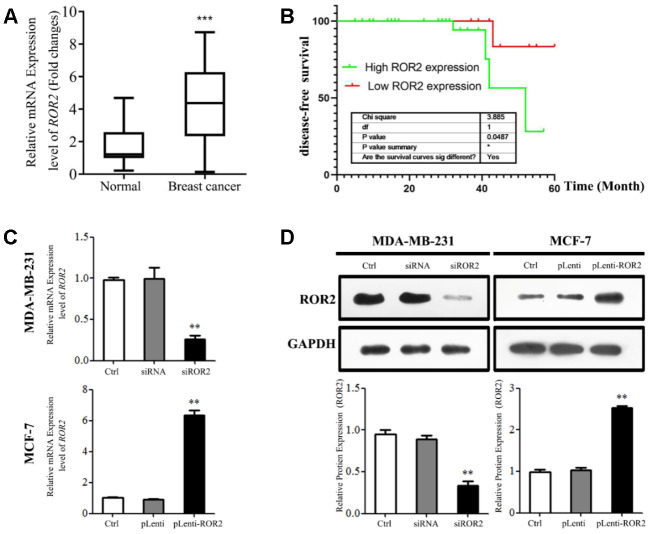
**High *ROR2* expression correlates with poor clinical outcome in BC patients.** (**A**) *ROR2* mRNA levels in 45 pairs of BC tissues compared with corresponding adjacent normal tissues. (**B**) Kaplan-Meier DFS curves for 45 BC patients classified according to *ROR2* mRNA levels. (**C**, **D**) ROR2 expression analyzed by qRT-PCR (**C**) and Western blotting (**D**) in MDA-MB-231 and MCF-7 cells transfected with siROR2 and pLenti-ROR2 plasmids. Image J software (version 1.48, NIH, USA) was used for the quantitative analysis of ROR2 protein levels analyzed by western blotting. Results are shown as means ± SD, n=3; *p<0.05, **p<0.01, ***p<0.001.

**Figure 2:**
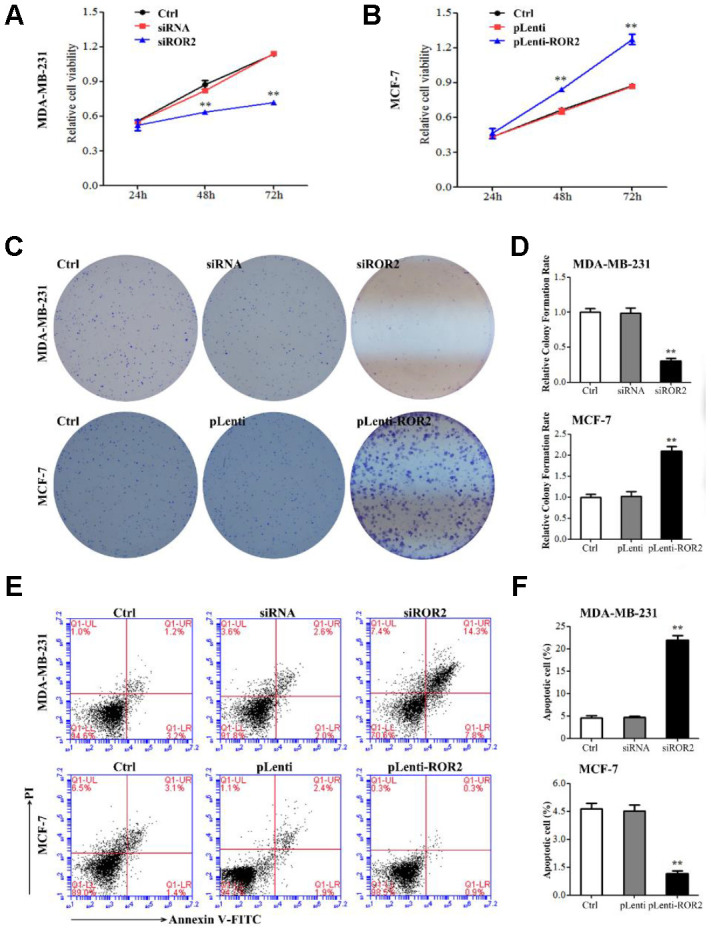
**ROR2 promotes BC cell proliferation *in vitro*.** (**A**, **B**) Cell proliferation analyzed by CCK-8 assay in MDA-MB-231 (**A**) and MCF-7 (**B**) cells transfected with ROR2 siRNA and overexpression plasmids. (**C**, **D**) Colony formation assay of ROR2 effect on MDA-MB-231 and MCF-7 cell proliferation. (**E**, **F**) Flow cytometry analysis of apoptosis of MDA-MB-231 and MCF-7 cells after siROR2 and pLenti-ROR2 transfection. Results are shown as means ± SD; n=3; *p<0.05, **p<0.01.

First, we analyzed *ROR2* mRNA levels in 45 BC tissues and adjacent pericarcinomatous tissues using qRT-PCR. As shown in Figure 1A, the *ROR2* gene expression was significantly increased in BC tissues compared with corresponding non-tumor tissues (*P<*0.01). As shown in Supplementary Table 1, the *ROR2* mRNA levels were not associated with age, differentiation, or TNM stage in BC patients. However, the increased *ROR2* expression was positively associated with the tumor diameter (P = 0.032). The Kaplan-Meier disease-free survival (DFS) curve revealed that BC patients with higher *ROR2* expression had a reduced DFS (Figure 2B). Analysis of *ROR2* mRNA levels in four different BC cell lines revealed that MCF-7 and MDA-MB-231 cells expressed the highest levels of *ROR2* compared to normal breast epithelial cell MCF-10A (Supplementary Figure 1). Therefore, we selected MDA-MB-231 cell line to knockdown ROR2, and MCF-7 cell line to overexpress ROR2 in subsequent experiments.

### ROR2 promotes BC cell proliferation in vitro

To analyze the ROR2 function in regulating BC cell proliferation, we suppressed the ROR2 expression in MDA-MB-231 cells using siRNA, and overexpressed ROR2 in MCF-7 cells using the ROR2-overexpression plasmid pLenti-ROR2. The endogenous ROR2 expression was effectively suppressed in siROR2 transfected MDA-MB-231 cells compared with cells transfected with control siRNA, while it was increased in pLenti-ROR2 transfected MCF-7 cells compared with control vector pLenti (Figure 1C, 1D). Proliferation of BC cells, analyzed by CCK-8 assay, was significantly inhibited in MDA-MB-231 cells transfected with siROR2, while it was increased in MCF-7 cells transfected with pLenti-ROR2 (Figure 2A, 2B). Furthermore, the colony formation ability of MDA-MB-231 cells was reduced by siROR2, but it was increased in MCF-7 cells by pLenti-ROR2 (Figure 2C, 2D). Flow cytometry analysis showed that ROR2 suppression increased apoptosis of MDA-MB-231 cells, while ROR2 overexpression decreased apoptosis ofMCF-7 cells (Figure 2E, 2F). These results suggest that ROR2 inhibits apoptosis and promotes proliferation of BC cells.

### ROR2 regulates expression of apoptosis-related genes in BC cells

**Figure 3:**
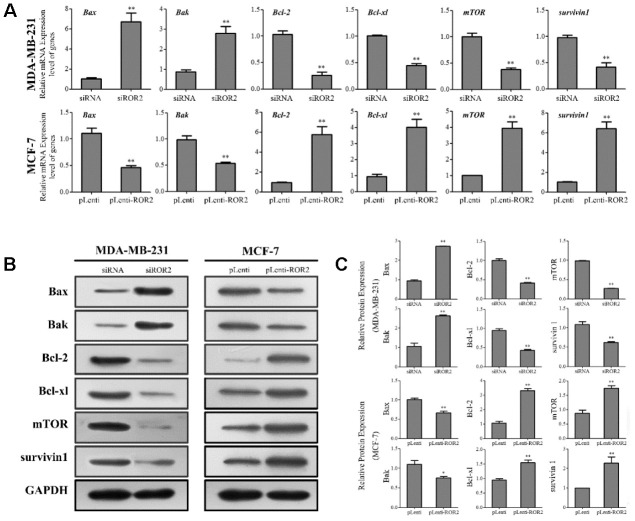
**ROR2 regulates expression of apoptosis-related genes in BC cells.** (**A**) qRT-PCR of *Bax, Bak, Bcl-2, Bcl-xl, mTOR* and *survivin 1* in MDA-MB-231 and MCF-7 cells after siROR2 and pLenti-ROR2 transfection. (**B**, **C**) Western blotting of Bax, Bak, Bcl-2, Bcl-xl, mTOR and survivin 1 in MDA-MB-231 and MCF-7 cells after siROR2 and pLenti-ROR2 transfection. Results are shown as means ± SD; n=3; *p<0.05, **p<0.01.

Our results showed that gene and protein levels of the pro-apoptotic markers Bax and Bak were increased in MDA-MB-231-siROR2 cells, and decreased in MCF-7-pLenti-ROR2 cells. In contrast, gene and protein expression of the anti-apoptotic markers BCL-2 and BCL-xl was decreased in MDA-MB-231-siROR2 cells, but increased in MCF-7-pLenti-ROR2 cells (Figure 3A–3C). Of note, the levels of mTOR and surviving-1 were decreased in MDA-MB-231-siROR2 cells and increased in MCF-7-pLenti-ROR2 cells (Figure 3A–3C). Collectively, these results suggest that ROR2 promotes BC cell proliferation by regulating expression of apoptotic genes.

### ROR2 induces PI3K/AKT signaling in BC cells

**Figure 4:**
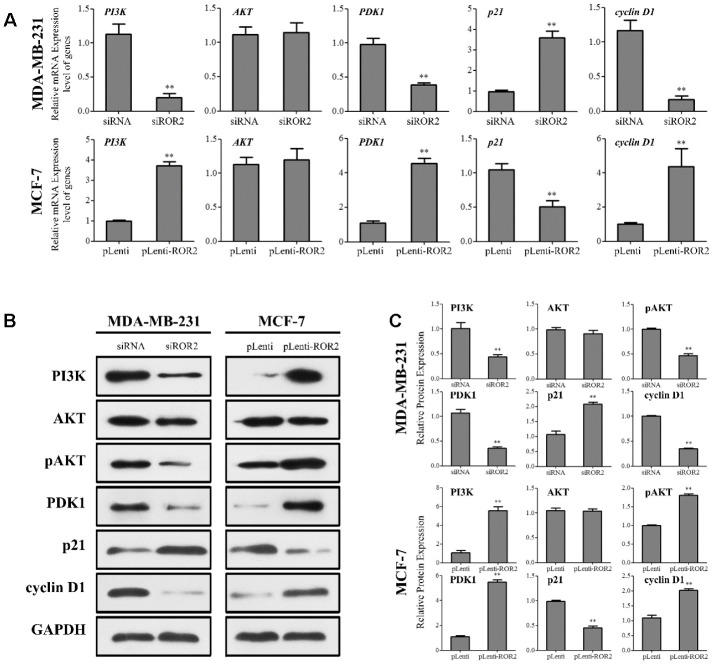
**ROR2 induces PI3K/AKT signaling in BC cells.** (**A**) qRT-PCR of *PI3K, AKT, PDK1, p21,* and *cyclin D1* in MDA-MB-231 and MCF-7 cells after siROR2 and pLenti-ROR2 transfection. (**B**, **C**) Western blotting of PI3K, AKT, pAKT, PDK1, p21, and cyclin D1 in MDA-MB-231 and MCF-7 cells after siROR2 and pLenti-ROR2 transfection. Results are shown as means ± SD; n=3; *p<0.05, **p<0.01.

Next, we investigated whether ROR2 regulates the PI3K/AKT signaling pathway in BC cells. ROR2 suppression reduced the protein levels of PI3K and phosphorylated AKT (p-AKT), while ROR2 overexpression increased the protein levels of PI3K and p-AKT (Figure 4A–4C). Furthermore, expression of the downstream genes of the PI3K/AKT pathway, PDK1 and cyclin D1 was reduced, while the expression of p21 was induced in MDA-MB-231 cells after transfection with siROR2. In contrast, the protein levels of PDK1 and cyclin D1 were induced, while p21 was reduced in MCF-7 cells transfected with pLenti-ROR2 (Figure 4A–4C). These results indicate that ROR2 activates the PI3K/AKT signaling in BC cells.

### ROR2 promotes BC tumorigenesis in vivo

**Figure 5:**
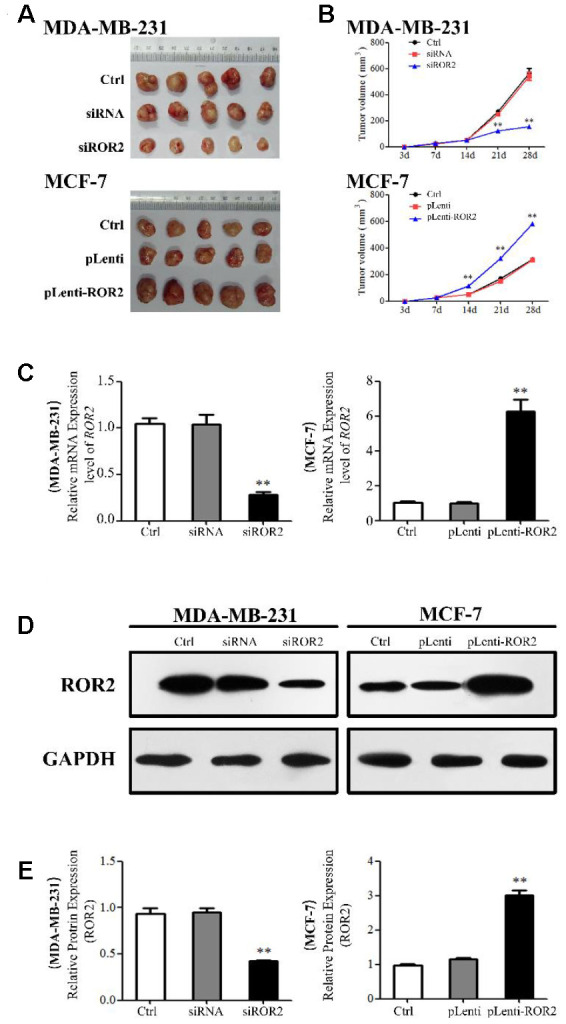
**ROR2 promotes BC tumorigenesis *in vivo*.** (**A**, **B**) Tumor growth in mice implanted with BC xenografts with suppressed (**A**) and overexpressed (**B**) ROR2. (**C**) qRT-PCR of *ROR2* in ectopic tumors. (**D**, **E**) Western blotting of ROR2 protein expression in ectopic tumors. *p<0.05, **p<0.01, *n*=6.

**Figure 6:**
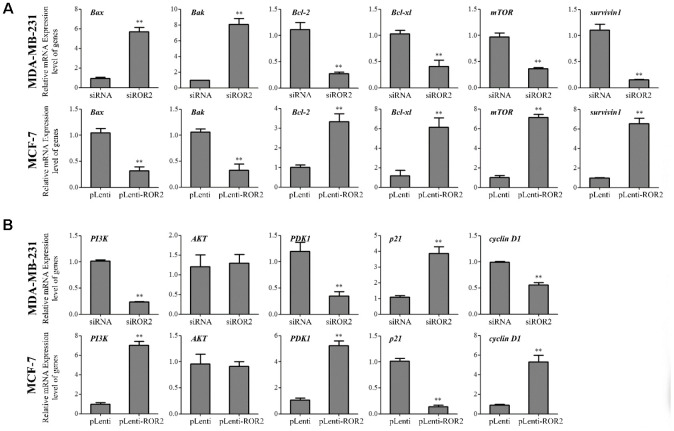
**ROR2 induces PI3K/AKT signaling *in vivo*.** (**A**) qRT-PCR of *Bax, Bak, Bcl-2, Bcl-xl, mTOR,* and *survivin 1* in MDA-MB-231 xenografts with ROR2 knockdown, and in ROR2-overexpressing MCF-7 xenografts. (**B**) qRT-PCR of *PI3K, AKT, pAKT, PDK1, p21,* and *cyclin D1* in the above tumors. Results are shown as means ± SD; n=3; *p<0.05, **p<0.01.

A xenograft model was established in mice implanted with MDA-MB-231 and MCF-7 cells to investigate the role of ROR2 in BC tumorigenesis *in vivo*. Four weeks after mice injections, the tumor volumes were significantly reduced in the MDA-MB-231-siROR2 group, compared with control or scrambled siRNA groups. In contrast, the tumor volumes were markedly increased in the MCF-7-pLenti-ROR2 group compared with control or pLenti vector groups (Figure 5A, 5B). The ROR2 expression in siROR2-formed tumor tissues was significantly lower than in control or siRNA groups, while the ROR2 expression in pLenti-ROR2-formed tumor tissues was higher than in control or pLenti vector groups (Figure 5C–5E). Moreover, expression of the apoptotic genes *Bax, Bak, Bcl-2, Bcl-xl, mTOR,* and *surviving-1*, and the PI3K/AKT signaling genes *PI3K, AKT, PDK1,* *p21*, and *cyclin D1* followed the same pattern as in the *in vitro* assays (Figure 6A, 6B). Together, these results indicate that ROR2 promotes BC tumor growth by regulating the expression of apoptotic and PI3K/AKT signaling genes.

## DISCUSSION

The receptor tyrosine kinase ROR2 is an important regulator in human cancers, serving as a tumor activator or an oncogene [3, 11, 15, 16]. The ROR2 expression is increased in breast cancer tissues compared to corresponding pericarcinomatous tissues [2, 12, 13]. In addition, ROR2 promotes the Wnt-mediated signaling in several types of cancer, including melanoma and colon cancer [10, 17].

In the present study, we also observed an increased expression of ROR2 in breast cancer tissues. Importantly, the increased ROR2 expression correlated with a poor prognosis in BC patients, suggesting that ROR2 has an important role in promoting BC cell proliferation. A previous study indicated that ROR2 might serve as an independent prognostic factor for squamous/adenosquamous carcinoma and gallbladder adenocarcinoma patients, and that ROR2 suppression might inhibit squamous carcinoma and gallbladder adenocarcinoma growth [13]. The ROR2 expression was increased also in human non-small cell lung cancer, and positively correlated with advanced TNM stage [5, 18]. In addition, ROR2 overexpression promoted renal cancer cell proliferation and activated the PI3K/AKT signaling pathway [15, 19]. In this study, we show that down-regulation of ROR2 significantly inhibits breast cancer cell proliferation *in vitro*, and BC tumor growth *in vivo*, and that the mechanism involves the phosphatidylinositol 3-kinase (PI3K)/AKT signaling pathway.

Targeting deregulated signaling pathways in human cancer has been a potentially effective approach in cancer therapy [1, 11, 20]. The PI3K/AKT signaling pathway, which is frequently deregulated in human cancers [21, 22], regulates multiple cellular processes, including cell proliferation, apoptosis, and cell migration [23–25]. A previous study has shown that some compounds exert their inhibitory effect on BC cell proliferation and growth through regulating the EGFR/PI3K/AKT axis [26]. Down-regulation of ROR2 decreased thyroid cancer cell proliferation and invasion *via* suppression of the PI3K/AKT signaling activation [27]. Our study demonstrates that ROR2 suppression reduces BC cell proliferation and tumor growth *in vitro* and in *vivo*, and induces apoptosis of BC cells by regulating the PI3K/AKT pathway. Furthermore, our results indicate that ROR2 overexpression activates PI3K, leading to AKT phosphorylation and activation, followed by up-regulation of PDK1 and cyclin D1, and down-regulation of p21, and resulting in the increased survival and proliferation of BC cells.

In conclusion, our results show that the expression of ROR2 is increased in human breast cancer tissues, and correlates with the DFS rates in BC patients. Knock-down of ROR2 suppresses BC cell proliferation and induces apoptosis *in vitro* and *in vivo*, while overexpression of ROR2 promotes BC cell survival and proliferation. In addition, down-regulation of ROR2 inhibits activation of the PI3K/AKT signaling pathway. Together, these data indicate that ROR2 acts as an oncogenic gene in BC, and suggest that the ROR2/PI3K/AKT regulatory network might contribute to breast cancer progression. Thus, ROR2 might serve as a novel biomarker for BC diagnosis, and a potential therapeutic target for BC therapy.

## MATERIALS AND METHODS

### Tissue specimens

A total of 45 pairs of BC tissues and paracancerous samples were collected from BC patients who had a mastectomy. The paracancerous tissues were taken 5 cm from the cancer tissues; all tissues were immediately stored at -80 °C. The paired adjacent non-tumor tissues were confirmed to have no tumor cells through pathological analysis. The patients did not receive radiotherapy or chemotherapy before the surgery. A written informed consent was obtained from all patients before the study. For the disease-free survival (DFS) study, each patient saw her doctor for a follow up assessment every 3 months in the first year, every 6 months in the second year, and annually after the second year. Dates of relapse were obtained from in-patient or outpatient records or from patients' families.

### Cell culture

Human breast cancer cell lines were obtained from the American Type Culture Collection (ATCC; Manassas, VA). Cells were cultured in HyClone^TM^ High-Glucose DMEM medium (Thermo, Cat. SH30243.01B, USA), supplemented with 10% fetal bovine serum (FBS; Gibco, Cat. 10099-133, USA) and 1% penicillin-streptomycin solution (Thermo, Cat. 15140122, USA), and incubated in a humidified chamber at 37°C supplemented with 5% CO_2_.

### Plasmid construction and RNA transfection

The ROR2 interfering plasmid siROR2 (Target sequence 5’-GGAAUAAGCAGAAGGCAU CTT-3’) and control interfering plasmid siRNA (Traget sequence 5’-UUCUCCGAACGUGUCACGUTT-3’) were obtained from General Biosystems (Anhui, China); the ROR2 overexpression plasmid pLenti-ROR2 and control plasmid pLenti were obtained from Applied Biological Materials (Jiangsu, China). About 1 × 10^6^ cells were seeded in 6-well plates and cultured for 24 h. Cells were transfected with 2 μg plasmid using Lipofectamine 2000 (Invitrogen, Carlsbad, CA, U.S.A.) in a serum-free medium in accordance with the manufacturer’s instructions. After 4 h, serum-free medium was changed to a complete medium containing 10% FBS. The transfection efficiency was more than 85%. Transfected cells were cultured with medium containing 1 μg/ml puromycin for 48 h, and further cultured in a medium containing 5 μg/ml puromycin to construct stable transfected cell lines for *in vivo* experiments.

### CCK-8 proliferation assay

Transfected cells were cultured in a medium containing 10% FBS for 24 h. About 2 × 10^4^ transfected cells were seeded in 96-well plates. Cell Counting Kit-8 (CCK-8; EnoGene, China) was used to evaluate the growth of breast cancer cells, according to the kit protocol. After incubation with CCK-8 at 37°C for 2-4 h, the absorbency was measured at 450 nm using Thermo Scientific Microplate Reader (Thermo MK3, U.S.A.).

### Colony formation assay

Transfected cells were seeded into 6-well plates at 400 cells/well, and incubated in a humidified chamber at 37°C with 5% CO_2_ for 1-2 weeks. To visualize and count the colonies, the colonies were fixed with methanol and stained with 0.5% crystal violet (Sigma, U.S.A.). Colony numbers were quantified by counting colonies that contained more than 25 cells observed under an inverted microscope. The experiments were performed in triplicates.

### Flow cytometry

Cell apoptosis was determined using Annexin V-FITC/PI (Propidium Iodide) apoptosis detection kit (EnoGene, China) in accordance with the manufacturer’s instructions. Transfected cells were harvested, washed with cold PBS, and re-suspended in binding buffer at a concentration of 1 × 10^6^ cells/ml at a final volume of 100 μL. Cells were then incubated with Annexin V-FITC (5 μL) and PI (5 μL) on ice. Each sample was re-suspended in 500 μL of binding buffer, and analyzed using a FACScan (BD, Biosciences, U.K.).

### RNA extraction and qRT-PCR

Total RNA was extracted using TRIpure reagent (Aidlab, China) and quantified using a NanoDrop2000 (Thermo Scientific, U.S.A.). 2 μg of RNA were used for reverse transcription reaction and cDNA synthesis using M-MLV Reverse Transcriptase (TaKaRa, Japan). The resulting cDNA products were then used as templates for PCR amplification. EvaGreen Express 2× qPCR MasterMix-ROX (abm, China) was used for qRT-PCR. The conditions of thermal cycling were as follows: 94 °C for 2 min, followed by 40 cycles at 94 °C for 20 s, 60 °C for 20 s. Samples were measured in triplicates and normalized to GAPDH. All primers were synthesized by Generay Biotech (Shanghai, China); the primer sequences (5’to 3’) are shown in Supplementary Table 2. TianLong medtl™ TL998-IV (TianLong, China) was used for qRT-PCR and data collection. 2^−ΔΔCT^ method was used to analyze the relative fold changes.

### Western blot analysis

Proteins were extracted using RIPA buffer, and protein concentration was measured using BCA Kit (EnoGene, Nanjing, China). Proteins were separated by 10% SDS-polyacrylamide gel and transferred onto a PVDF membrane (Bio-Rad, USA). The membranes were blocked with 5 % skimmed milk at room temperature for 2 h, and washed in TBS-Tween 20. Subsequently, the membranes were incubated with anti-ROR2 (Biovision, Cat. 6702-100), anti-Bax (EnoGene, Cat. E11-0132C), anti-Bak (EnoGene, Cat. E11-0131C), anti-Bcl-2 (EnoGene, Cat. E10-30077), anti-Bcl-xl (EnoGene, Cat. E90209), anti-mTOR (EnoGene, Cat. E11-7156B), anti-survivin 1 (Biorbyt, Cat. orb394299), anti-PI3K (Biorbyt Cat. orb137259), anti-AKT (bioss, Cat. bs-0115R-1), anti-pAKT (bioss, Cat. bs-12458R-1), anti-PDK1 (EnoGene, Cat. E10-30154), anti-p21 (Abcam, Cat. ab215971-p21), anti-cyclin D1 (Abcam, Cat. ab185241 - Cyclin D1), and control anti-GAPDH (EnoGene, Cat. E12-052) antibodies at 4 °C overnight. After washing in TBS-Tween 20, the membranes were incubated with secondary antibodies conjugated to horseradish peroxidase (HRP, EnoGene) at 37 °C for 1 h. Protein bands were detected using ECL Western Blotting System (Millipore, MA, U.S.A.) and visualized using image analyzer (DKSH, USA). The immunoblot signal was quantitated with ImageJ software and the values were normalized to the GAPDH band density.

### Xenograft tumor model

Eight-week-old BALB/c nude mice with an average weight of 20 g were obtained from Changzhou Cavens Experimental Animal Co., Ltd (Jiangsu, China). Tumors were established by injection of 5×10^6^ transfected MAD-MB-231 or MCF-7 cells in 100 μl of PBS into the subcutaneous flanks of nude mice. Tumor dimensions were measured using electronic calipers. Tumor volumes were calculated by the formula: L ×W^2^ × 0.5, where L is the largest diameter and W is the perpendicular diameter. 28 days after implantation, the mice were euthanized using 20% CO_2_ exposure for 10 min. The tumors were resected for further analysis. The animal protocol followed the guidelines of the animal care committee of the Nanjing First Hospital, and all procedures were approved by the ethics committee of Nanjing First Hospital.

### Statistical analysis

All assays were performed in triplicates and repeated at least three times. Results are shown as mean ± SD. The statistical analyses for p values were obtained using SPSS18.0 software (SPSS, Inc., Chicago, IL, USA). The comparison of different groups was analyzed using the unpaired, two-tailed Student’s t-test. Values of p < 0.05 were considered statistically significant.

### Ethical statement

This work was approved by Nanjing Medical University.

## Supplementary Material

Supplementary Figure 1

Supplementary Tables
