# Supplementary material for: ROR2 knockdown suppresses breast cancer growth through PI3K/ATK signaling
Source: Aging (Albany NY). 2020 Jul 2;12(13):13115–27. doi: 10.18632/aging.103400 (PMC7377870; doi:10.18632/aging.103400)
Supplement: Supplementary Figure 1 [file aging-12-103400-s001..pdf]

SUPPLEMENTARY FIGURE

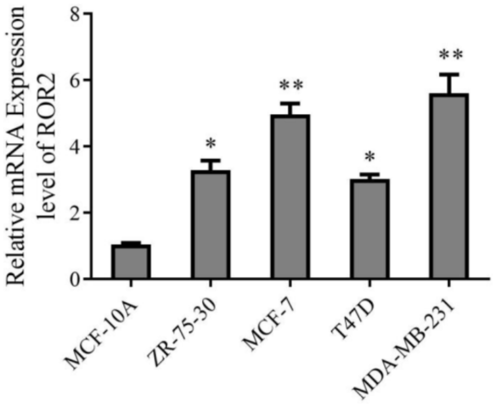

Supplementary Figure 1. The expression of ROR2 in different BC cell lines.*p<0.05, **p<0.01.
