# Supplementary material for: ROR2 knockdown suppresses breast cancer growth through PI3K/ATK signaling
Source: Aging (Albany NY). 2020 Jul 2;12(13):13115–27. doi: 10.18632/aging.103400 (PMC7377870; doi:10.18632/aging.103400)
Supplement: Supplementary Tables [file aging-12-103400-s002..pdf]

SUPPLEMENTARY TABLES

Supplementary Table 1. Clinicopathological characteristics in BC patients and expression of ROR mRNA.

Variables	No. of patients	High expression of ROR2	Low expression of ROR2	P value
Age(year)				0.311
≥ 55	32	23	9	
< 55	13	11	2	
Diameter				0.032
≥2(cm)	29	25	4	
<2(cm)	16	9	7	
Differentiation				0.533
High	27	20	7	
Low/Middle	18	14	4	
TNM Stage				0.096
I-II	23	15	8	
III	22	19	3	

*p<0.05

Supplementay Table 2. Primer sequences used for qRT- PCR analysis.

No.	Gene symbol		Forward Primer(5’ to 3’)	Reverse Primer(5’ to 3’)	Length (bp)
1	GAPDH	Human	5’-CCTCTGACTTCAACAGCGACAC-3’	5’-CTGTTGCTGTAGCCAAATTCGT-3’	121
2	ROR2	Human	5’-AGTGTCCCGGACTTCAGGT-3’	5’-CCTTGCAGTGCAGAATTGCC-3’	173
3	Bax	Human	5’-CCAGAGGCGGGGTTTCAT-3’	5’-TGAGACACTCGCTCAGCTTC-3’	116
4	Bak	Human	5’-CCCTCTGCTTCTGAGGAGCA-3’	5’-GCTAGGTTGCAGAGGTAAGG-3’	147
5	Bcl-2	Human	5’-GATGGGATCGTTGCCTTATGC-3’	5’-GTCTACTTCCTCTGTGATGTTGT-3’	103
6	Bcl-xl	Human	5’-GGGGTCGCATTGTGGCCTTT-3’	5’-GCTCTAGGTGGTCATTCAGG-3’	129
7	mTOR	Human	5’-CCGCTCTCTGAAGAACCTCC-3’	5’-AGGTTCTCGGGTCAGATCCA-3’	82
8	survivin 1	Human	5’-CTTTCTCAAGGACCACCGCATC-3’	5’-CTTCCAGCTCCTTGAAGCAGAA-3’	161
9	PI3K	Human	5’-TCCCTGGCCTTCACTGTCTA-3’	5’-GGGGCTGCACATGTCCTT-3’	117
10	AKT	Human	5’- ACTGTCATCGAACGCACCTT -3’	5’-CTCCTCCTCCTCCTGCTTCT-3’	108
11	PDK1	Human	5’-GTGGTTTATGTACCATCCCATC-3’	5’-GGTAAACACCTCTGTTGGCATG-3’	100
12	p21	Human	5’-GACTGTGATGCGCTAATGGC-3’	5’-GTCACCCTCCAGTGGTGTCT-3’	90
13	cyclin D1	Human	5’- TGAAGTTCATTTCCAATCCG-3’	5’- GGGTCACACTTGATCACTCT-3’	145
14	GAPDH	Mouse	5’- TGTGGAAGGGCTCATGACCACA-3’	5’-GCAGGGATGATGTTCTGGGCAG-3’	117
15	ROR2	Mouse	5’-TTGGGCCTCTTCCGTGAGGTAT-3’	5’-AGGACCACACCGTAGGACCAGA-3’	146
16	Bax	Mouse	5’-TTCTTCCGGGTGGCAGCTGACA-3’	5’-CTTTAGTGCACAGGGCCTTGAG-3’	112
17	Bak	Mouse	5’-GGAGGTCTTTCGAAGCTACGTT-3’	5’-GGCAAGTTGTCCATCTCGGGGT-3’	93
18	Bcl-2	Mouse	5’-GGCTGGGGATGACTTCTCTCGT-3’	5’-GTTCCTCCACCACCGTGGCAAA-3’	113
19	Bcl-xl	Mouse	5’-GGAGAGCGTTCAGTGATCTAAC-3’	5’-CACGATGCGACCCCAGTTTAC -3’	119
20	mTOR	Mouse	5’-TCCGCTACTGTGTCTTGGCA-3’	5’-GCTCGCGGATCTCAAAGACC-3’	117
21	survivin 1	Mouse	5’-TAAGGAATTGGAAGGCTGGGAA-3’	5’-ATCTGCTTCTTGACAGTGAGGA-3’	96
22	PI3K	Mouse	5’- TGGTTAGACTTGGATCCTTGGG -3’	5’- TTGGCCCTGGGCTTTTGTAA -3’	162
23	AKT	Mouse	5’- CCGCCTGCCCTTCTACAACC-3’	5’- CGAGCCTCTGTGTAGGGTCC-3’	143
24	PDK1	Mouse	5’-CCAGCACTCCTTATTGTTCGGT-3’	5’-TTGTCTGTCCTGGTGATTTCGC-3’	197
25	p21	Mouse	5’-CGTCCTCCCAAGATAGCCGA-3’	5’-CAGGCGCGGTCATACTTGTA-3’	152
26	cyclin D1	Mouse	5’-GAGGAGCAGAAGTGCGAAGA-3’	5’-GGCTCTTCTTCAAGGGCTCC-3’	88
